# Precursor turbulent inflow dataset for large eddy simulation of a semi-idealized European generic city

**DOI:** 10.1016/j.dib.2024.110467

**Published:** 2024-04-23

**Authors:** Usman Shaukat, Knut Erik Teigen Giljarhus

**Affiliations:** Department of Mechanical and Structural Engineering and Materials Science, University of Stavanger, Stavanger, Norway

**Keywords:** Precursor simulation, Michelstadt city, Atmospheric boundary layer wind tunnel, Turbulent inflow condition, Urban wind environment, Semi-idealized city, Pedestrian wind comfort

## Abstract

This data article provides high-quality turbulent inflow boundary data with a high spatial and temporal resolution of a very rough atmospheric boundary layer (ABL) wind tunnel, which can be applied as the large eddy simulation (LES) inflow condition on the Michelstadt test cases. A high-quality LES of the WOTAN wind tunnel of the Environmental Wind Tunnel Laboratory (EWTL) was conducted using OpenFOAM software, and data is stored at a plane at 1000Hz frequency at the end of the roughness elements. This data serves as the turbulent inflow boundary condition, offering computational fluid dynamics (CFD) researchers a cost-effective means to simulate the benchmark Michelstadt test cases for LES validation. This data will be utilized to perform high-quality LES, which are pivotal in bridging the research gap in understanding the intricate nature of wind dynamics in a realistic urban environment.

Specifications Table

**Table:** 

Subject	Engineering, Environmental Engineering
Specific subject area	Large Eddy Simulation (LES), Urban Wind Environment, Pedestrian Wind Comfort, CFD simulation of Wind
Type of data	Raw, Analyzed, Simulated Data
Data collection	Data was collected by performing a high-quality LES of an atmospheric boundary layer (ABL) wind tunnel with turbulence-generating structures using OpenFOAM-v2206 CFD software. The data was extracted near the outlet plane using the post-process utility of OpenFOAM-v2206 to provide inlet conditions for subsequent simulation of flow over the generic city. The simulations were carried out using high-performance parallel computing CPUs at the University of Stavanger campus.
Data source location	Institution: University of Stavanger City/Town/Region: Rogaland, Stavanger Country: Norway
Data accessibility	Repository name: Zenodo Data identification number: 10.5281/zenodo.10656886 Direct URL to data: https://zenodo.org/records/10656886

## 1 Value of the Data

• The accuracy of LES is dependent on the inflow conditions, and obtaining good turbulent inflow conditions for LES is challenging in urban wind engineering [1].
• This high-quality turbulent inflow wind dataset is extracted at 1000Hz frequency with high spatial resolution for a very rough ABL wind tunnel of CEDVAL- LES Michelstadt test cases [2].
• This dataset can be used as an inflow boundary condition for researchers to validate LES on benchmark Michelstadt test cases [2], [3], [4], [5], [6], [7].
• Michelstadt test cases offer time series of wind data and were originally designed to validate the LES model, establishing new CFD guidelines for LES [8].
• The researchers can utilize this data by using a TimeVaryingMappedFixedValue inlet boundary condition in OpenFOAM-v2206 (or later versions), eliminating the need for precursor simulations and thus reducing the high computational costs of LES.

## 2 Background

With the availability of high-performance computers, LES is becoming popular in wind engineering applications as they potentially give better accuracy than simulations based on Reynolds-Averaged Navier Stokes (RANS) equations [1]. LES has been applied to numerous real-world applications [9], [10], [11]. However, there is still a shortage of well-established validation guidelines for real-world cases [1]. Therefore, wind tunnel measurements were conducted at the EWTL of the University of Hamburg and referred to as Michelstadt test cases [2]. These measurements became part of the CEDVAL-LES reference database (available at https://cloud.uni-hamburg.de/s/bjNeP9GpH36mKKk), offering the time-resolved data for different types of neutrally stratified boundary layer flows for validating LES studies.

Since improper inflow conditions in LES can alter the local pressure and flow around the buildings, it is essential to have accurate inflow turbulent boundary conditions for conducting high-quality LES. Therefore, this study performs LES of the development section of a very rough turbulent boundary layer wind tunnel, ``Complexity-3'' [2]. The researchers can reuse this data for Michelstadt test cases to validate their LES model, saving the cost of carrying out precursor simulation for appropriate inflow boundary conditions.

## 3 Data Description

The recorded dataset contains the velocity time-series data at the plane, including high spatial and temporal resolution for the turbulent inflow boundary condition. The dataset is compressed in the zip format and is named “inlet.zip.” The total compressed size of the data is approximately 39.5GB. The dataset is stored in the OpenFOAM boundary data format to facilitate compatibility with OpenFOAM's processing capabilities. The dataset structure is as follows:

• The “inlet.zip” file has the inlet directory, which contains a total of 120000 individual subdirectories and one single file named ``points.''
• These individual subdirectories' names represent the precise time and are written with an increment of 0.001 s time step while the “points” file contains the spatial coordinates.
• Each subdirectory of time is equipped with a “U” file detailing a three-dimensional velocity vector written inside it that corresponds to the spatial coordinates in the “points” file, ensuring the precise mapping of the fluid velocity at each time step.

**Fig. 1:**
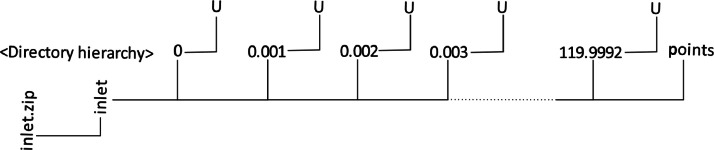
Hierarchy of directory inside the inlet.zip file.

The U and points files of the dataset are in ASCII format. Fig. 1 illustrates the directory layout for better understanding.

The inlet plane data is in a model scale of 1:225, similar to the wind tunnel specifications for Michelstadt cases, but it can be scaled to full scale. For details, see the “How to reuse?” section.

• Data validation

**Fig. 2:**
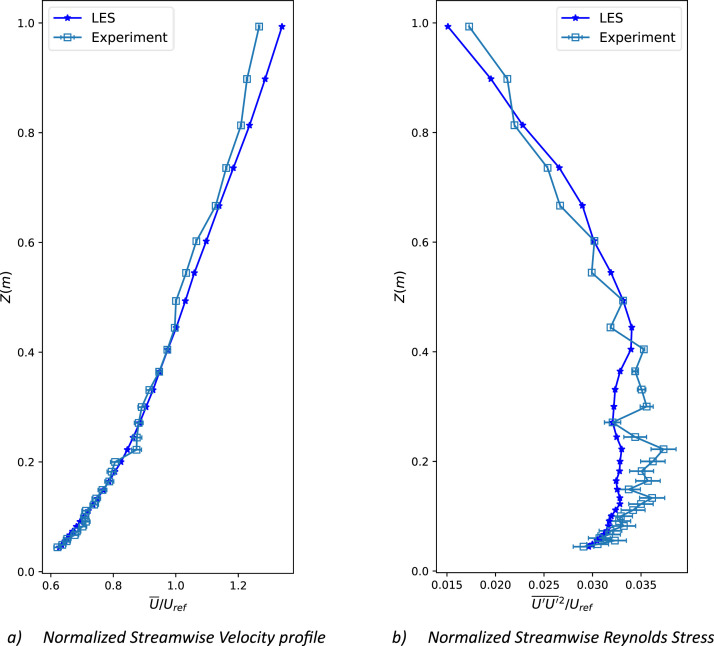
LES simulated vertical profile comparison against the wind tunnel experiment in the middle in front of the roughness element of the wind tunnel.

The inflow turbulence dataset was verified against the wind tunnel measurements for dataset quality assurance. Fig. 2a and b compare vertical profiles of the normalized mean streamwise velocity and normalized streamwise Reynolds stress at the lateral center in front of the roughness elements against the experimental findings. The reproduced wind profile follows the power law with a power index of 0.27 and a roughness length of 1.5, which is characteristic of a very rough turbulent boundary layer at EWTL [2]. The turbulence was also well reproduced with an average relative error of 3.1 %, as can be visualized from the Reynolds stress profile Fig. 2b. This error is within the modeling of smaller subgrid scales that are usually not adequately resolved in LES.

## 4 Experimental Design, Materials and Methods

• Computational Domain

**Fig. 3:**
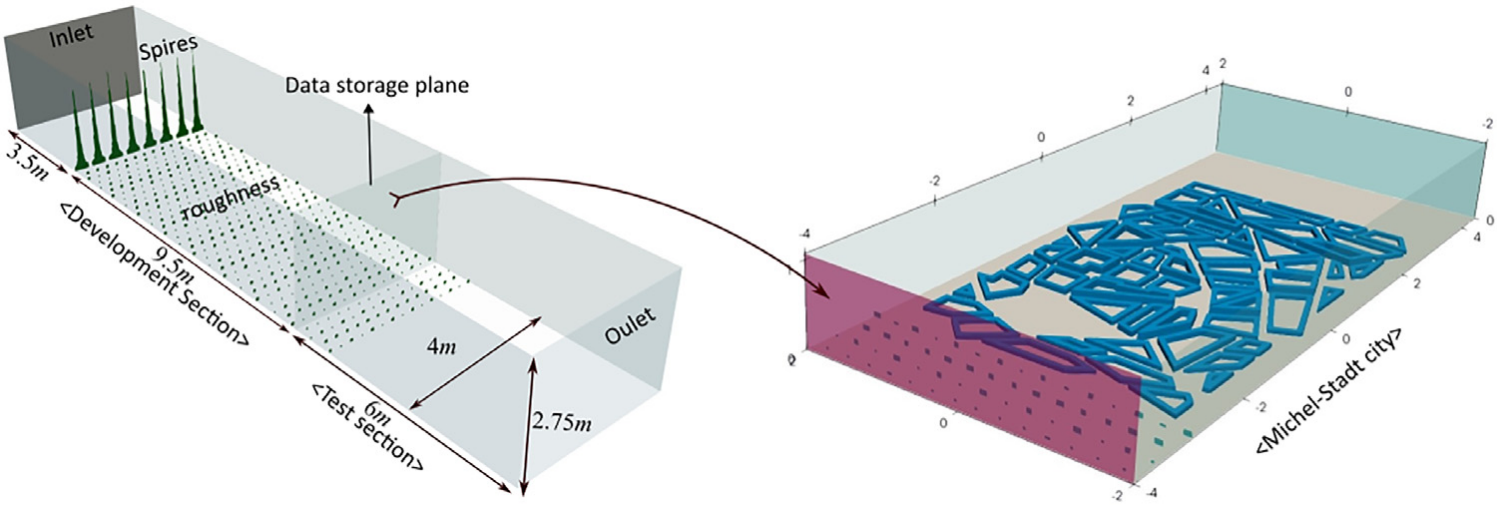
ABL wind tunnel setup is shown on the left, and the right figure illustrates an example of how to use the turbulent inlet data on the Michelstadt test city case.

For obtaining high-quality inflow turbulence data for the Michelstadt benchmark cases, the development section of the computational wind tunnel was created by replicating the very rough ABL of the WOTAN wind tunnel having a model scale of 1:225 at the EWTL of the University of Hamburg [2]. The computational domain was extended 19m streamwise, 4m spanwise, and 2.75m vertically. The experimental wind tunnel was cloned properly by introducing eight vertical spires at a 3.5m distance from the inlet, followed by a series of thirty-five rows of roughness elements with a 0.3m separation distance between them. The center-to-center lateral spacing between the elements was 0.28m. The roughness element consisted of two sizes, 40mm and 80mm. The arrangement of these elements is shown in Fig. 3. More details about the experimental setup can be found in the original wind tunnel experiment [2].

**Fig. 4:**
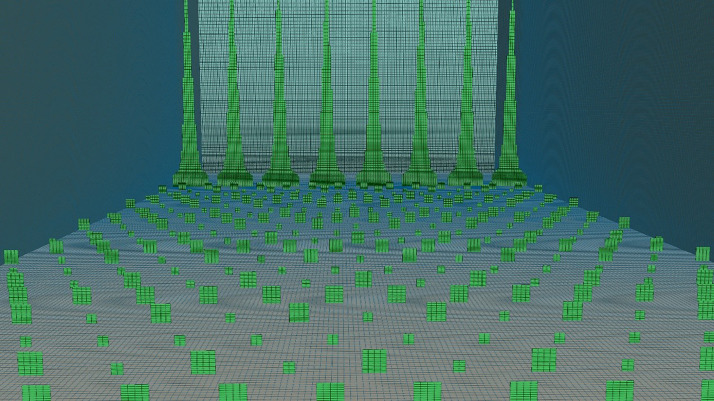
Illustration of surface mesh and arrangement of roughness elements and spires.

The mesh consists of 14 million hexahedral cells generated with the blockMesh utility in OpenFOAM. It maintains uniform distribution in longitudinal, transverse, and vertical directions up to; 0.08 m; above this, a stretching factor of 1.03 was applied in the vertical direction. As a result, the smallest roughness element had two cells horizontally and six cells vertically, resulting in an aspect ratio of 3 below the roughness elements, as illustrated in Fig. 4.

• Numerical Setup

The simulation utilized the standard Smagorinsky model with a Smagorinsky constant (*C*_s_) of 0.12 in OpenFOAM-v2206. A second-order numerical scheme was employed for temporal discretization, ensuring a maximum Courant number below 1 for all time steps. The simulation ran for 120 s. Spatial discretization employs a second-order blended scheme with 5 % upwind for reducing numerical instability. The wind speed at the reference height of 0.444 m was $U_{ref}=\;4.55\;m/s$.

• Data extraction method for inflow condition

The extraction plane for extracting the inflow turbulence data is shown in Fig. 3. Velocity data is extracted from this plane at a frequency of 1000Hz for a duration of 120s using OpenFOAM's post-processing utility, covering the entirety of the simulation time. It is recommended for the user to apply boundary conditions once the flow is fully established, typically after 20s, corresponding to approximately six bulk flow-through cycles in the wind tunnel. This utility stores the data in the OpenFOAM boundary condition format.

• How to reuse?

**Fig. 5:**
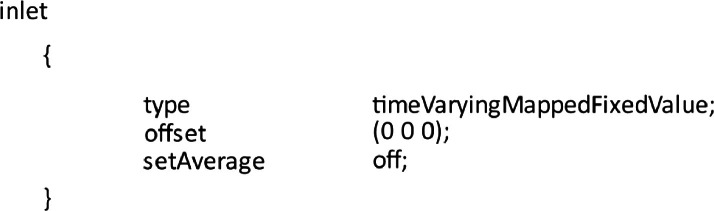
Example of defining inlet boundary condition in U file for OpenFOAM cases.

Users can extract the files inside the boundary data subdirectory of the constant directory of the OpenFOAM case (the case to be simulated). The inlet boundary condition can be specified in the U file of 0 folder using the TimeVaryingMappedFixedValue boundary condition of the OpenFOAM-v2206 version, as shown in Fig. 5. Users are required to consult the OpenFOAM documentation for other versions. Fig. 3 on the right illustrates the graphical interpretation of how the inflow condition will be mapped onto the other Michelstadt cases.

To scale a simulation model, which is based on the Michelstadt test case with a scale of 1:225, to a full scale, users should follow these steps:

1. Start with the “point” file containing the spatial coordinates present inside the “boundaryData” directory, see Fig. 1. These coordinates should be scaled by a factor of 225 to convert them into full-scale dimensions.
2. The time data subdirectories, recorded at a 1000 Hz frequency, should also be converted to full scale. Timestep increment is adjusted using the relation $\Delta T^{f}=\left(\Delta T^{m}/L_{m}\right)\times L_{f}$. Where, $\Delta T^{m}$ is the time step for the model scale, *L*_m_ is the model scale reference length (1 m), and $L_{f}$ is the full-scale reference length (225 m).
3. Finally, rename these timestep directories from the model scale to reflect their corresponding full-scale values, as calculated in step 2.

## Limitations

The data only includes the turbulent inflow profile in OpenFOAM format. The reader is required to change the format if they wish to use it on other software.

## Ethics Statement

The current work does not involve human subjects, animal experiments, or any data collected from social media platforms.

## CRediT authorship contribution statement

**Usman Shaukat:** Software, Conceptualization, Investigation, Formal analysis, Writing – original draft. **Knut Erik Teigen Giljarhus:** Conceptualization, Investigation, Supervision, Writing – review & editing.

## Data Availability

Precursor turbulent inflow dataset for large eddy simulation of a Michelstadt city (Original data) (Zenodo).
